# A machine learning approach to facilitate parasitic egg identification in a conspecific brood parasite

**DOI:** 10.1098/rspb.2025.2085

**Published:** 2025-11-26

**Authors:** Anna E. Hughes, Lisandrina Mari, Jolyon Troscianko, Václav Jelínek, Tomas Albrecht, Michal Šulc

**Affiliations:** ^1^ Department of Psychology, University of Essex, Colchester CO4 3SQ, UK; ^2^ Department of Biological and Environmental Science, University of Jyväskylä, 40014 Jyväskylä, Finland; ^3^ Institute of Vertebrate Biology Czech Academy of Sciences, 603 65 Brno, Czech Republic; ^4^ Centre for Ecology and Conservation, University of Exeter, Penryn TR10 9FE, UK; ^5^ Department of Zoology, Charles University, 128 44 Prague, Czech Republic

**Keywords:** cognition, artificial intelligence, intraspecific nest parasitism, egg phenotype, colour, pattern

## Abstract

Avian brood parasitism offers an excellent system for studying coevolution. While more common than interspecific parasitism, conspecific brood parasitism (CBP) is less studied owing to the challenge of detecting parasitic eggs. Molecular genotyping accurately detects CBP, but its high cost has led researchers to explore egg appearance as a more accessible alternative. Barn swallows (*Hirundo rustica*) are suspected conspecific brood parasites, yet parasitic egg detection has largely relied on subjective human assessment. Here, we used UV–visible photographs of genetically confirmed non-parasitized barn swallow clutches and simulated parasitism to compare the accuracy of human assessment with supervised machine learning models. Participants and models completed two classification tasks, identifying parasitic eggs from either six or two options. Both humans and the ‘leave-one-clutch-out’ model performed better than chance, with accuracies of 72 and 87% (humans) and 76 and 92% (models). An improved ‘leave-one-egg-out’ model achieved 97% accuracy, greatly exceeding human performance, likely by integrating more visual information, with egg dimensions being the most important trait, followed by colour and spotting pattern. We present a complete and accessible pipeline for replicating our supervised models, offering a powerful tool to identify parasitic eggs in other species also, and advance research on the evolution of egg phenotypes.

## 1. Introduction

Avian brood parasitism represents an alternative reproductive strategy wherein parasitic females lay some or all of their eggs in nests belonging to other females. This phenomenon has been documented in 365 species, with 109 species being interspecific obligate brood parasites that consistently lay their eggs in nests of different species [1]. The interactions between interspecific brood parasites and their hosts have become a major focus for investigating coevolutionary processes in nature [2].

Conspecific brood parasitism (CBP) accounts for the remaining 70% of brood parasites and involves individuals occasionally laying eggs in the nests of others within the same species [3]. Despite its prevalence, CBP remains relatively understudied, largely owing to the challenges of detecting it in the wild. Unlike the eggs of obligate brood parasites such as the common cuckoo (*Cuculus canorus*), which, even when highly mimetic, can usually be easily distinguished from host eggs [4], identifying conspecific parasitic eggs is more difficult because they typically resemble the host’s eggs very closely.

Early studies on CBP relied solely on field observations, such as detecting multiple eggs laid in a single day or noting differences in egg appearance [5–10]. However, later research introduced more precise methods, like protein and genetic fingerprinting, to confirm CBP [11–14]. While these genotyping techniques are highly accurate, they remain costly, time-consuming and technically demanding. Consequently, researchers have explored the potential of using egg phenotype and modern analytical methods to identify parasitized clutches and specific parasitic eggs. This non-invasive, low-cost approach also offers the advantage of increasing sample sizes, as genetic fingerprinting may not always be feasible, particularly when predation occurs before blood sampling or when unfertilized eggs without viable DNA are present.

The identification of CBP based on egg phenotype is theoretically possible only in species where individual females lay eggs that are more similar to each other than to those of other females. This pattern, characterized by low variation within clutches and high variation among clutches, appears to be widespread in birds [11,15,16]. Two groups in particular may benefit from this phenomenon: hosts of brood parasites, which can more easily recognize and eject parasitic eggs that differ from their own [17–19], and colonially nesting birds, which might otherwise mislay eggs or misdirect incubation and nest defence behaviours [20,21].

The reliability of using egg phenotype to identify parasitized clutches and specific parasitic eggs remains, however, a debated issue. Much of the research on this topic has focused on egg size parameters in waterfowl, which are frequent conspecific brood parasites [22]. Eadie [23] proposed an automatic unsupervised method based on maximum Euclidean distance (MED) that identifies parasitized clutches by detecting eggs that deviate significantly from the others in the same clutch—more than would typically be expected from a single female’s eggs. His thesis, along with a study by Pöysä *et al.* [24], demonstrated that this method is reliable for identifying parasitized clutches of common goldeneyes (*Bucephala clangula*). However, other studies that have replicated this approach in various waterfowl species have reported mixed results, indicating that the method’s reliability varies between species, and they advise using it with caution [25–29]. A more nuanced approach was proposed by Eadie *et al.* [30], who suggested categorizing clutches into two groups: (1) clutches that can be reliably classified as parasitized or non-parasitized, and (2) clutches where the model’s classification is uncertain. While this more conservative method improved the accuracy, the authors still recommend combining its results with observational or molecular techniques for the best outcomes. Recent technological and computational advances have enabled the use of supervised machine learning methods to investigate visual cues in ecological research [31,32]. However, their application to the study of egg phenotypes remains limited, with only a few studies using these techniques [16,33]. Similarly, deep learning methods have been applied in only a small number of recent studies [34,35], despite their demonstrated success in other areas of animal identification [32,36,37], which is likely owing to their limited interpretability and the requirement for relatively large, genetically validated training datasets [38].

Over 30 years ago, Møller’s pioneering studies revealed CBP in the barn swallow (*Hirundo rustica*). He identified this behaviour by noting the appearance of two eggs in active nests during the same day [7] and by finding eggs in experimental, non-active nests [39]. Since Møller never directly observed the parasitizing individuals, he relied on egg appearance to identify parasitic eggs and even to speculate on the identity of females that laid them. However, it is well documented that the last eggs laid by various species, including barn swallows, often differ from the other eggs in the clutch (reviewed in [40]), which increases the risk of misidentification when using this method alone [41,42]. Therefore, the use of egg appearance for accurately identifying parasitized clutches and specific parasitic eggs in barn swallows still requires further validation.

In this study, we investigated whether egg phenotype can be reliably used to identify parasitized clutches and parasitic eggs in the barn swallow (hereafter swallow), a widely assumed conspecific brood parasite (but see also recent findings on the extremely low level of brood parasitism in this species [14]). Using a labelled dataset of swallow eggs that were molecularly assigned to their genetic mothers, we simulated brood parasitism and tested the ability of human participants, both with and without expert knowledge of swallow egg appearance, to identify foreign (‘parasitic’) eggs within ‘host’ clutches. Additionally, we applied analytical techniques to quantify phenotypic traits, including size, shape, spotting pattern and colour from UV–visible photographs and compared the identification accuracy of the unsupervised and supervised tools with that of human participants. The high accuracy of the supervised model used in this study was previously demonstrated for common cuckoo eggs [16]. In the present study, we adapted it to explore its potential for advancing research on conspecific brood parasitism. Beyond our specific findings on barn swallow eggs, we provide a complete machine learning pipeline to facilitate future studies investigating egg phenotypes in other brood parasitic species.

## 2. Material and methods

### (a) Study population

We collected data during the 2020 and 2021 breeding seasons at four farms in the villages Stará Hlína (49°02′21.4″ N, 14°49′06.8″ E), Břilice (49°01′14.4″ N, 14°44′15.3″ E), Lužnice (49°3'25.3" N, 14°46'11.4" E) and Lomnice nad Lužnicí (49°4'7.7" N, 14°42'36.7" E) in southern Bohemia, Czech Republic. Here, swallows breed inside cattle barns, nesting on walls, beams, or lamps, or in crevices near the ceiling. They start arriving in late March, and females usually start laying eggs in April and May. From May to July, we conducted four mist-netting sessions and ringed all adults with a unique combination of aluminium and plastic coloured rings. All individuals were sexed, measured, weighed and photographed, and a venipuncture blood sample (approx. 20 μl) was taken. Most active nests were found before egg laying and monitored daily during the egg-laying period and at 2–5 day intervals during the nestling stage. Chicks were ringed and blood sampled (approx. 10 μl) at 9 days old, and unhatched eggs were collected for embryonic tissue samples. All blood and embryonic tissue samples were stored in 96% ethanol. We selected 54 clutches, ensuring that each was laid by a different female and that the same female laid all eggs within a clutch. Parentage was verified by genotyping all parents and offspring with 17 microsatellite markers using Geneious Prime^®^ 2024.0.3 (GraphPad Software) and Cervus 3.0.7 (Field Genetics Ltd). Further details on molecular assignment are available in the electronic supplementary material and in Jelínek *et al.* [14], which presents a comprehensive maternity analysis in our study population from 2010 to 2021.

### (b) Egg photography and image analysis

Avian eggs, including those of swallows, reflect both visible and ultraviolet (UV) light [43], and birds are known to use UV signals for egg recognition [44,45]. Therefore, we took human-visible and UV-spectrum photographs of swallow eggs within the first three days after clutch completion using a full spectrum Samsung NX 1000 camera with a Nikkor EL 80 mm lens. Visible-light photographs were taken through a Baader UV–IR blocking filter (Baader Planetarium, Mammendorf, Germany; 420–680  nm), and UV photographs with a Baader UV pass filter (320–380 nm). All eggs of a given clutch were placed on the side on a dark board inside a white nylon light tent (50 × 50 × 50 cm, Fomei, China; see electronic supplementary material) and photographed together in the shade, at the same angle and from the same distance to minimize lighting variability during the day [46]. Despite this uniform protocol, we detected a small quadratic effect of time of the day (but no effect of calendar day) on the colour and luminance variables extracted from our photographs (see electronic supplementary material). Although we do not believe this influenced our final results, we recommend restricting image acquisition to a specific time of the day to reduce the noise introduced by natural light [46]. RAW images were calibrated by polytetrafluoroethylene grey standards (3 and 97% reflectance). Exposure settings were adjusted accordingly with lighting conditions, yet the International Organization for Standardization (ISO) value was set constant at 400 and aperture *f*/8. Image calibration and pattern, colour and shape analysis were performed in ImageJ [47] using the Multispectral Image Calibration and Analysis (MICA) Toolbox [48,49]. All images were rescaled to the scale of the smallest image (30 pixels mm^−1^).

For colour analysis, we used a custom ImageJ script (K. Szala 2025, personal communication) that incorporates functions of the MICA Toolbox [48] to measure the brightness and colour of egg spots and background independently. Spots were detected using the Phansalkar thresholding method [50] with Gaussian blur correction for uneven illumination [51]. We applied a 50 pixel radius for thresholding and a 2048 pixel Gaussian blur. To prevent incorrect thresholding in darker egg edges, we reduced the selection area around each egg by 3% of its width. We extracted reflectance values for red (R), green (G), blue (B) and ultraviolet (UV) channels, as well as brightness values, for both the spots and the background of each egg. Additionally, using the MICA Toolbox [48], we also calculated pixel proportions across ten luminance levels (ranging from 0 to 1 in 0.1 increments) to capture a more detailed description of the overall luminance of the entire egg.

For pattern analysis, we applied granularity analysis to quantify pattern energy at different spatial frequencies [48,52]. Since pattern energy alone does not differentiate between dark spots on a light background and light spots on dark background, we also calculated the skewness of the pattern, which quantifies the asymmetry of the pattern luminance distribution. Additional information and the code for calculating skewness can be found in Šulc *et al.* [16]. Pattern energies and skewness were calculated across the whole egg and separately for the blunt pole, sharp pole and middle section to measure within-egg pattern variability.

Additionally, we used a custom ImageJ script (K. Szala 2025, personal communication) to calculate average spot size, the percentage of egg surface covered by spots, and three measures of pattern dispersion. Pattern dispersion parameters provided insights into how spots are distributed along the egg’s long axis. By analysing pattern coverage for three sections (blunt pole, sharp pole and middle), we calculated the mean and standard deviation (s.d.), and coefficient of variation (s.d./mean × 100) of pattern dispersion.

For shape analysis, we used the MICA Toolbox [48] to calculate egg length, maximum width, volume, surface area, ellipse deviation and ellipse aspect ratio (parameter *a* in [53]).

Since clutch size in our swallow population is typically five eggs, we selected photographs of five-egg clutches laid by different females as verified by molecular analysis (see below). We excluded low-quality images (blurry or unevenly illuminated), resulting in a dataset of 270 eggs from 54 females.

### (c) Preparing variables describing egg appearance

We collected colour, pattern, size and shape data from calibrated photographs of 270 swallow eggs (54 clutches, each containing 5 eggs). To avoid correlated variables in models, we performed principal component analyses (PCA) on different egg features and selected PCA components based on scree plot inspection [54]. Percentages of variation explained by these selected components are listed below.

*Colour and luminance data*. To perform a PCA for the colour features, we used average R, G, B, UV and brightness values for spots and background. For luminance, we conducted a PCA using the pixel proportions across ten luminance levels across the entire egg. From these analyses, three colour PCA components (explaining 95% of variance) and one luminance PCA component (explaining 41% of variance) were included in the final dataset.

*Pattern data*. Four PCA components for pattern features were included in the dataset. The first two components (explaining 54% of variance) were derived from a PCA of 12 pattern energy values and 12 skewness values, measured at multiple scales (from 1 to 0.0221 in steps of 1/√2) across the whole egg and within each of three egg segments. The remaining two components (explaining 92% of variance) were calculated from a separate PCA of average spot size, percentage of egg surface covered by spots and the mean, s.d. and coefficient of variance of pattern dispersion.

*Shape data*. The last PCA was conducted for egg length, maximum width, volume, surface area, ellipse deviation and aspect ratio. One shape PCA component (explaining 55% of variance) was included in the final dataset.

The final dataset included nine PCA components, referred to as egg phenotypic traits, which were used for further analyses.

### (d) Within- and between-clutch variance in egg appearance

For egg appearance to be a reliable indicator of parasitized clutches and parasitic eggs, the within-clutch variation must be lower than the between-clutch variation. To assess this, we first scaled all nine phenotypic trait variables to standardize distance calculations. To quantify within-clutch variance, we calculated the s.d. for each trait from all eggs within a clutch and averaged these s.d. values across all traits, providing an overall variance metric for each female. To quantify between-clutch variance, we calculated the average value of each phenotypic trait from all eggs in a clutch, effectively creating an ‘average’ egg of each clutch. We then calculated the s.d. for each phenotypic trait across all clutches and averaged these s.ds to generate a metric of between-clutch variance across all traits. To test whether within-clutch variance is indeed lower than between-clutch variance, we performed a one-sample *t*‐test, comparing the within-clutch variance metric (*n* = 54) against the test value representing between-clutch variance.

Additionally, we calculated Beecher’s information statistic (Hs) to quantify how well egg phenotype signals individual identity. This metric is particularly valuable as it allows comparisons across various studies, species and signature systems [55,56]. This analysis was conducted using the R package *IDmeasurer* [56]. To validate our findings, we compared the results from the real data with a control statistic generated by shuffling the ID labels [16].

### (e) Ranking phenotypic traits by their prediction accuracy

To assess which phenotypic traits are best at predicting parasitic eggs, we fitted a random forest model using the R package *randomForest* [57]. This model was applied to the entire dataset of 270 eggs and their associated female identities to determine the prediction accuracy of each phenotypic trait, measured by mean decrease in accuracy. Mean decrease in accuracy quantifies the reduction in accuracy when a specific variable is excluded, with higher values indicating greater importance for classification. The mean decrease in accuracy values was also used as weights to transform the phenotypic trait values to improve accuracy in identifying parasitized clutches and individual parasitic eggs in subsequent models.

We weighted each phenotypic trait by multiplying it with the corresponding mean decrease in accuracy value from the random forest model. Using weighted phenotypic traits yielded higher prediction accuracy of the unsupervised method compared with unweighted traits (see electronic supplementary material).

### (f) Identification of parasitized clutches

To assess whether egg appearance can be used to distinguish parasitized clutches from non-parasitized clutches, we adapted Eadie’s method [23,30] using the maximum Euclidean distance (MED). For each non-parasitized clutch in our dataset (*n* = 54), we calculated Euclidean distances between all possible egg pairs within a clutch (10 comparisons per clutch of 5 eggs) based on nine weighted egg phenotypic traits. Then, we calculated a mean distance for each egg by taking the average Euclidean distance of all the egg pairs it was involved in. The value of the most different egg was identified as the MED for that clutch. For parasitized clutches, we created 54 exemplar parasitized clutches by using slides from participant 1 in game 1 (see below for further details), and randomly removed one host egg from each clutch to standardize them to a total of five eggs per clutch. We then carried out the same process to calculate the MED for each clutch as for the non-parasitized clutches. Subsequently, we compared MED values between parasitized and non-parasitized clutches using a *t*‐test and assessed distribution overlap to evaluate this method’s effectiveness.

### (g) Identification of parasitic eggs

We used human assessment and two automatic methods to identify parasitic eggs in barn swallow clutches, all using a dataset of 270 eggs from 54 clutches. For human assessment, we created calibrated RGB images of all eggs in ImageJ by using the MICA Toolbox [48] and rescaled them to 10 pixels mm^−1^ to match the dimensions of commonly used computer screens. To simulate brood parasitism, we added a randomly selected swallow egg from a different female (the parasitic egg) to each five-egg clutch laid by the same female (host eggs), generating 14 310 unique combinations of parasitized clutches. We then randomly selected 1890 combinations, ensuring that each participant assessed all 54 host clutches with a randomly selected parasitic egg. This approach finally resulted in 1641 unique combinations of parasitized clutches, 114 combinations being duplicated twice and seven combinations being duplicated three times.

### (h) Human assessment

To evaluate human ability to discriminate a parasitic egg from five host eggs, we tested 105 participants, divided into three groups based on their experience with bird eggs. The first group consisted of 35 researchers or students with extensive recent experience handling barn swallow eggs (‘experienced with swallow eggs’). The second group included 35 researchers experienced with wild bird eggs but not swallow eggs (‘experienced with bird eggs’). The final group comprised 35 participants with no prior experience with wild bird eggs (‘inexperienced’).

**Figure 1.**
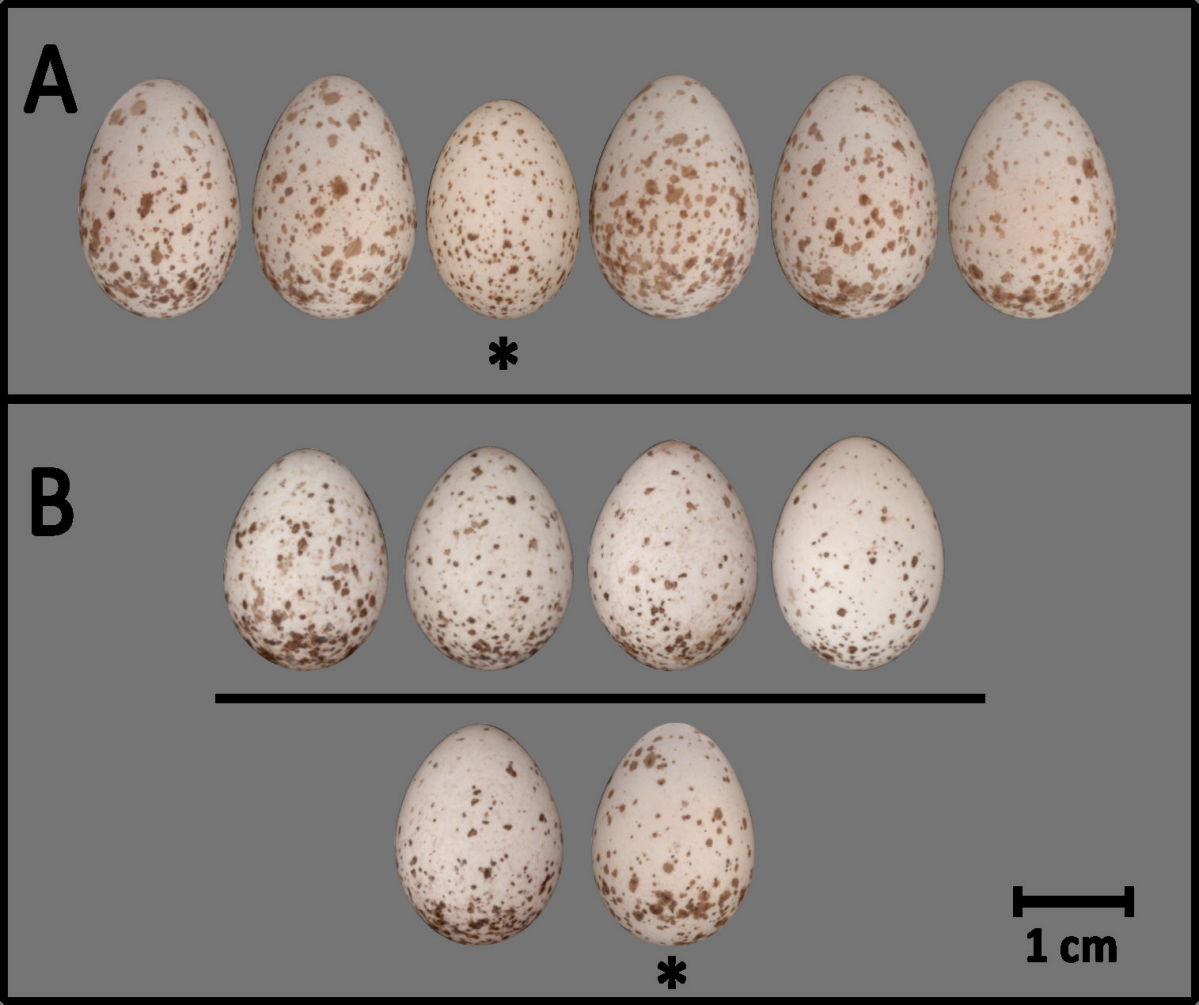
Example slides from the screen games. Both games included 54 slides, each displaying a six-egg clutch with five eggs laid by a single ‘host’ female and one by a different ‘parasitic’ female. In game 1 (A), participants visually identified the parasitic egg from all six eggs. In game 2 (B), participants chose only between two eggs (bottom row), while the four eggs above were confirmed as host eggs. Asterisks indicate the parasitic eggs. Additional slides for readers to test their ability to identify parasitic eggs are available in the electronic supplementary material.

We designed two online screen tests (hereafter games) using PsychoPy [58], with participants completing both games, with an interval of one to two months apart. Each game featured 54 slides displaying parasitized clutches on a grey background. In game 1, participants were tasked with selecting one egg that appeared most different from a randomly ordered set of six eggs (complete clutch of five eggs plus one parasitic egg), simulating a scenario where a researcher encounters a complete clutch without knowing the egg-laying order (figure 1A). In game 2, participants were presented with four randomly selected host eggs from a five-egg clutch in the top row and two eggs below—the remaining fifth host egg and a parasitic egg (figure 1B). Participants had to choose between the two bottom-row eggs, simulating a scenario where a researcher conducts daily nest checks and finds four eggs laid in the normal laying rhythm (i.e. one egg per day), but two eggs laid on the same day, with one of these presumed to be parasitic.

Every participant within a specific group received a unique set of parasitized clutches, generating 1890 combinations of parasitized clutches (54 slides × 35 participants). Participants with the same number (1–35) in each experience group received identical sets of parasitized clutches, allowing comparison across experience groups. Additionally, every participant received the same set of parasitized clutches in both games, enabling us to assess improvement from game 1 to game 2.

### (i) Automatic assessments

#### (i) Unsupervised classification: Euclidean distance analysis

We applied an unsupervised classification model for each game. In game 1, we calculated Euclidean distances based on nine weighted egg phenotypic traits for all 15 possible egg pairs in each six-egg parasitized clutch. The parasitic egg was identified as the one within a clutch with the highest mean egg distance (across all phenotypic traits), and we measured the model’s accuracy as the percentage correct assignment across all 1890 clutches. For game 2, a similar procedure was followed, except we compared only two candidate parasitic eggs, selecting the one with the greatest mean egg distance.

#### (ii) Supervised classification: same/different analysis

We used a supervised random forest model to classify egg pairs as either ‘same’ (laid by the same female) or ‘different’ (laid by two different females), the same approach as we previously implemented for identification of eggs laid by the common cuckoo [16]. The model was trained on a balanced dataset of 265 ‘same’ and 265 ‘different’ cases and validated using a ‘leave-one-clutch-out’ approach [59], meaning that for each tested egg, the training dataset consisted only of eggs laid by different females (*n* = 265). In the test phase, we compared each egg with all others, obtaining 72 630 comparisons (270 × 269) with two comparisons per egg pair (one for each egg as the target). Since we used only within-clutch comparisons from the same 1890 combinations of parasitized clutches as in human assessments, this resulted in 10 comparisons (2 × 5) for each egg within a clutch. As generating the training dataset involves stochastic processes, we repeated this procedure ten times, resulting in 100 comparisons for each egg within the parasitized clutch. This approach reduced the number of inconclusive identifications (i.e. where the model assigned the same number of ‘different’ classifications to multiple eggs) and improved overall accuracy.

In game 1, we compared all eggs within a parasitized clutch, identifying the parasitic egg as the one most frequently classified as ‘different’. In game 2, a similar procedure was followed, except we compared the two candidate parasitic eggs with the remaining four eggs and selected the one with the highest number of ‘different’ results. Finally, we calculated the model’s accuracy as the percentage of times an egg was correctly identified as ‘parasitic’. In rare cases, the model identified more than one egg as parasitic (i.e. same number of ‘different’ results); we considered these assessments ambiguous and did not include them in the final accuracy calculation.

For game 2, we additionally applied a random forest model that included, in the training dataset, eggs from the tested clutch that were laid in a regular daily sequence (i.e. one egg per day). We refer to this method as the ‘leave-one-egg-out’ approach. This analysis was designed for situations where researchers assume that these regularly laid eggs were produced by the host female and aim to identify which of the two additional eggs laid on the same day is parasitic. By incorporating known host eggs, we expected the model to improve the accuracy of assigning the remaining host egg and identifiying the parasitic egg. We did not apply this approach in game 1, as it was designed to reflect a real-life scenario where the laying order of eggs is unknown.

**Figure 2.**
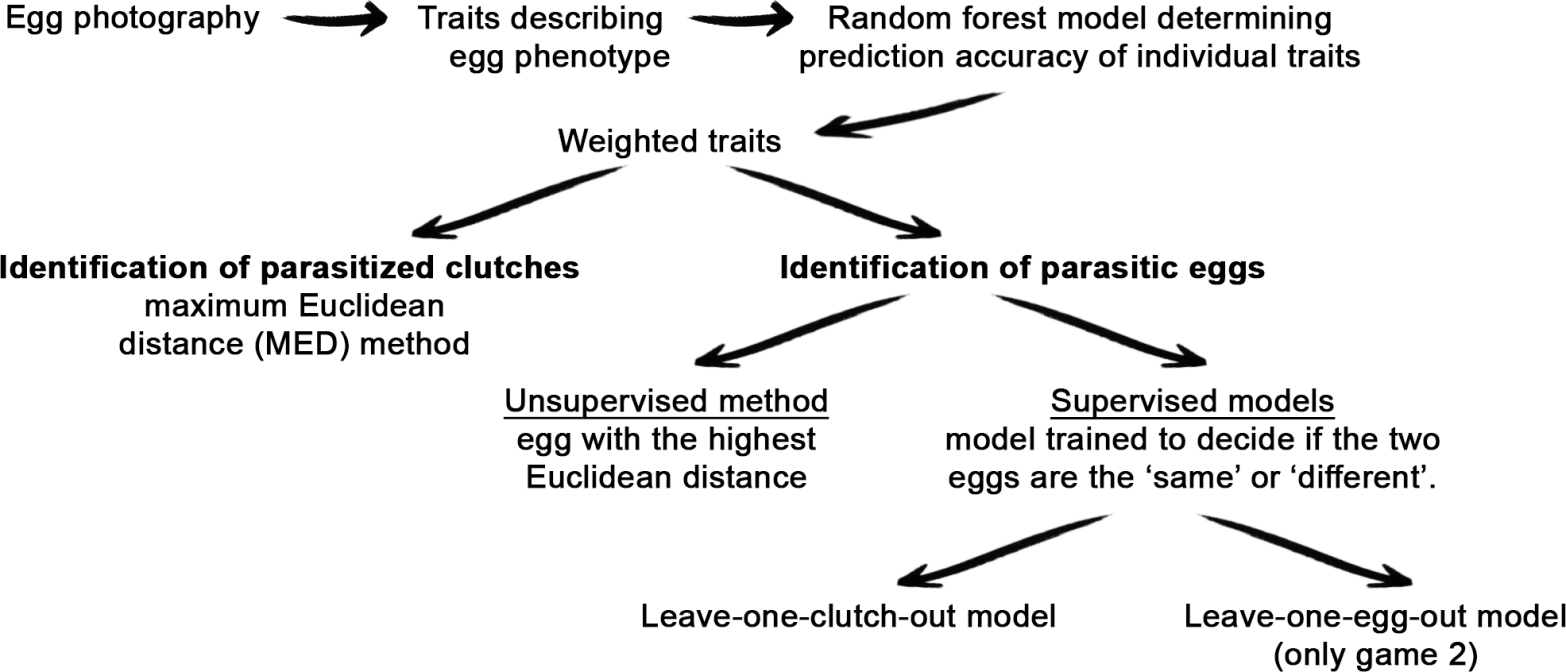
Workflow of methods used for the automatic identification of parasitized clutches and parasitic eggs. For further details, please refer to Material and methods.

The full workflow of methods used for the automatic identification of parasitized clutches and parasitic eggs is illustrated in figure 2.

### (j) Supplementary analyses

We also ran an unsupervised *k*-means clustering analysis and a supervised one-class support vector machine analysis. Both of these methods were relatively poor at discriminating between parasitic and host eggs, so we do not present them in the main manuscript, but full details can be found in the supplementary code.

## 3. Results

### (a) Within- and between-clutch variance in egg appearance

The mean within-clutch variance was 0.58 (s.d. = 0.16; range 0.30–1.06, all clutches can be seen in the electronic supplementary material). Overall, between-clutch variance (mean = 0.82, s.d. = 0.05; range = 0.76–0.91) was higher than within-clutch variance (one-sample *t*‐test, *t* = 26.02, d.f. = 53, *p* < 0.001). Beecher’s information statistic (a measure of how well egg phenotype signals individual identity) Hs = 0.93, considering only significant variables. This can be compared with a control Hs = 0.33, where the ID labels were randomly shuffled. Variation in the egg appearance is also graphically illustrated in the electronic supplementary material.

### (b) Ranking traits for prediction

PC1 for shape was the most important variable for egg classification, and the variables loading onto this PC were the length, maximum width, volume and surface area of the egg. The second most important variable was PC3 for colour, where the spot UV channel, the background UV channel and the spot brightness contrasted with the background visible channels (R and G in particular). The importance of all nine traits used for egg classification is summarized in table 1.

**Table 1.** Ranking of trait importance for egg classification by using a random forest model. The main principle component analysis (PCA) loadings are those that were greater than ±0.25*.*

variable	mean decrease in accuracy	main PCA loadings
PC1 shape	37.13	length, maximum width, volume, surface area
PC3 colour	32.06	spot UV channel, spot brightness and background UV channel versus background R and G channels
PC2 pattern	28.44	—
PC2 colour	22.48	spot R, G and B channel versus background B and UV channels and background brightness
PC1 colour	21.30	spot R, G, B and UV channels, background R, G, B and UV channels, spot and background brightness
PC1 pattern	20.44	—
PC1 luminance	19.73	luminance bands 0.1, 0.2, 0.3, 0.4, 0.5 versus 0.6 and 0.7
PC2 pattern 2	18.61	standard deviation and coefficient of variance of pattern dispersion
PC1 pattern 2	17.95	average spot size, percentage of the egg covered by spots, mean and standard deviation of pattern dispersion

### (c) Identification of parasitized clutches

**Figure 3.**
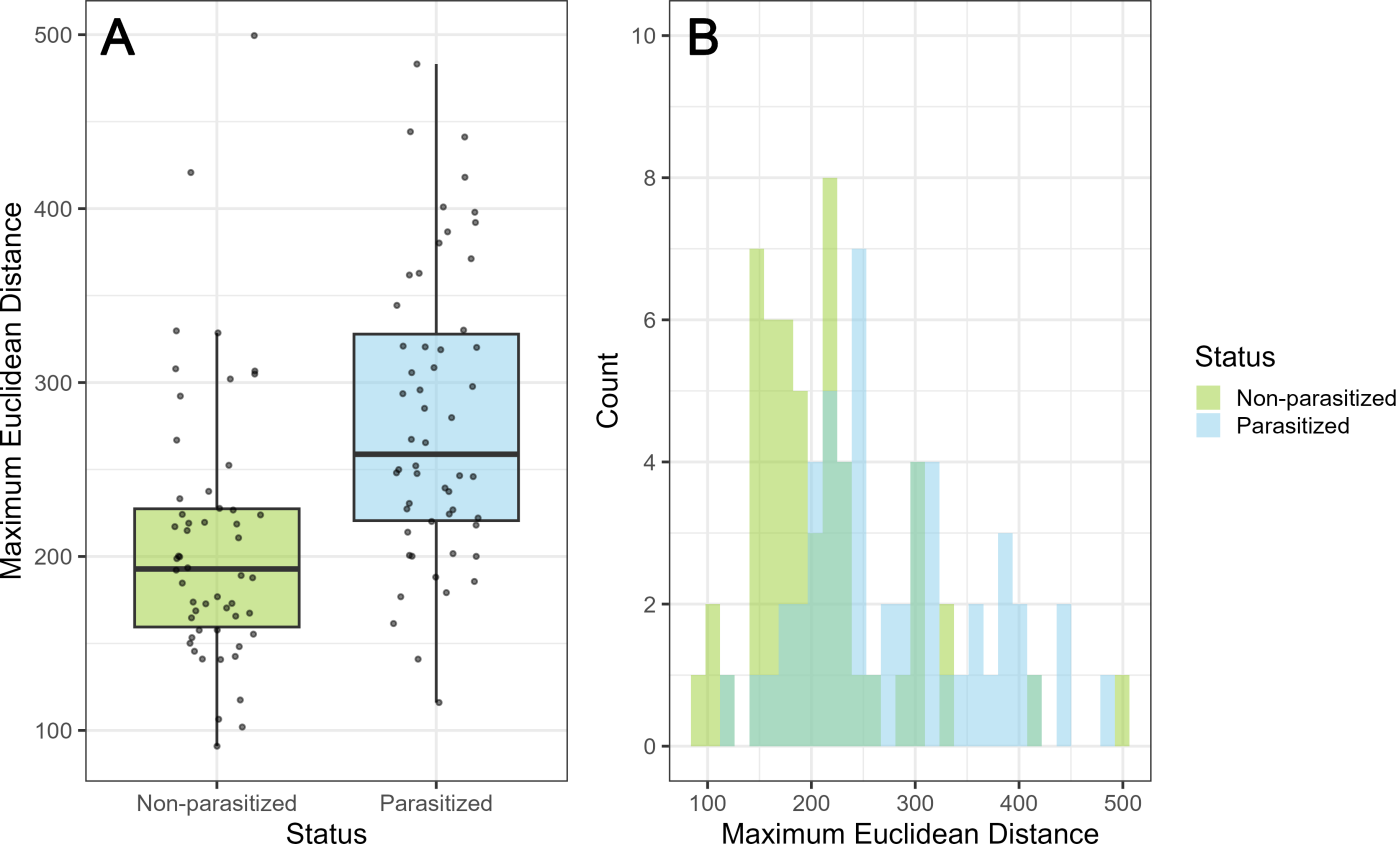
(A) Maximum Euclidean distances (MEDs) calculated for 54 non-parasitized and 54 parasitized clutches. (B) Frequency distribution of MED depicting the substantial overlap between non-parasitized and parasitized clutches.

The MED was significantly higher in parasitized compared with non-parasitized clutches (*t* = 4.51, d.f. = 104, *p* < 0.001; figure 3A), with 96% (52 out of 54) of non-parasitized clutches showing an MED below 350. This suggests that clutches with an MED above this threshold are likely to be parasitized. However, there was a considerable overlap between the MED distributions of parasitized and non-parasitized clutches (figure 3B), and the majority of parasitized clutches (42 out of 54) showed MED values below this threshold. MED on its own, therefore, cannot reliably distinguish parasitized and non-parasitized clutches.

### (d) Identification of parasitic eggs

#### (i) Human assessment: game 1

**Figure 4.**
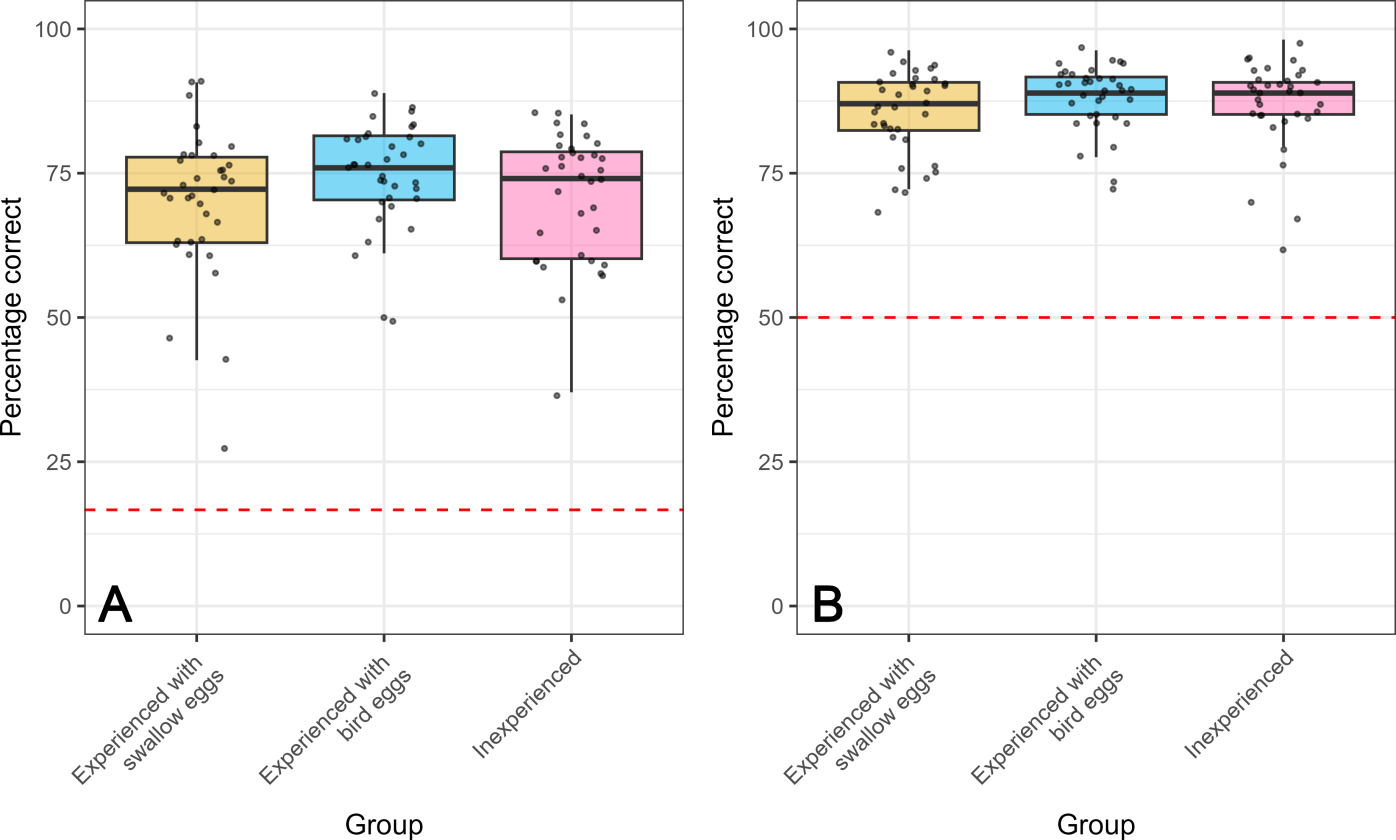
(A) Percentage of parasitic eggs correctly assigned by three differently experienced groups of human participants in game 1. Since participants were choosing from six-egg options, the red line denotes a level of chance, i.e. 16.7% correct answers. (B) Percentage of parasitic eggs correctly assigned by three differently experienced groups of human participants in game 2. Participants were choosing from two-egg options, so the red line denotes a 50% level of chance.

Overall, participants performed relatively well on this task, achieving an average accuracy of 71.9%, which was significantly above chance (Wilcoxon signed rank test compared with a mean of 9: *V* = 5565, *p* < 0.001; figure 4A). Accuracy did not differ among the three experience groups (one-way ANOVA: *F*_2,102_ = 1.7, *p* = 0.188), indicating that expertise did not affect the performance of the human assessment. The mean time taken on each decision was 10.04 s (s.d. = 13.97).

#### (ii) Human assessment: game 2

Participants performed better on the second game, where they selected between only two eggs, compared with the first game (paired-sample *t*‐test: *t* = −16.851, d.f. = 104, *p* < 0.001). In the second game, participants achieved a mean accuracy of 86.8%, which was significantly higher than chance (Wilcoxon signed rank test compared with a mean of 27: *V* = 5565, *p* < 0.001; figure 4B). As in the first game, accuracy did not significantly differ among the three experience groups (one-way ANOVA: *F*_2,102_ = 1.31, *p* = 0.274). The mean time taken on each decision was 7.40 s (s.d. = 19.67).

Participants who performed well in game 1 also tended to do well in game 2 (Pearson’s correlation coefficient: *r* = 0.59, *t* = 7.45, d.f. = 103, *p* < 0.001). Additionally, participants who spent more time playing the game generally achieved higher accuracy (average time across games, *r* = 0.37, *t*(103) = 4.01, *p* < 0.001).

### (e) Automatic method: unsupervised classification

In game 1, our unsupervised method based on weighted Euclidean distances predicted the correct parasitic egg with 62.2% accuracy, which was significantly lower than the accuracy of human participants (Wilcoxon signed rank test, *V* = 4979, *p* < 0.001). The unsupervised approach performed better in game 2 by identifying correctly parasitic eggs in 86.5% of cases, which was comparable to human performance (Wilcoxon signed rank test, *V* = 3365, *p* = 0.06).

### (f) Automatic method: supervised classification

First, we applied the ‘leave-one-clutch-out’ approach for both games. In game 1, the model correctly identified 1417 of 1890 parasitic eggs, and in 456 clutches another egg was flagged as the parasitic egg. This resulted in a 75.7% accuracy rate, which was significantly better than human assessment (Wilcoxon signed rank test, *V* = 1742, *p* < 0.001). In the remaining 17 clutches, one host egg was incorrectly flagged as parasitic alongside the parasitic egg, making it impossible to confidently identify the parasitic egg based on this supervised method.

In game 2, the model identified a single egg as parasitic in 1877 out of 1890 clutches, of which 1720 were truly parasitic eggs, and 157 were host eggs, yielding a 91.6% accuracy, which was higher than the accuracy of human participants (Wilcoxon signed rank test, *V* = 585, *p* < 0.001). In the remaining 13 clutches, the model did not allow a definite decision to be made as both target eggs were flagged as parasitic at the same rate.

When we applied the ‘leave-one-egg-out’ approach for game 2 (see Methods), the model identified a single egg as parasitic in 1887 out of 1890 clutches, of which 1838 were truly parasitic eggs, and 49 were host eggs. Only the remaining three clutches showed ambiguous results as both target eggs were flagged as parasitic at the same rate. This model, therefore, showed a 97.4% accuracy rate, which again significantly outperformed the accuracy of human assessment (Wilcoxon signed rank test, *V* = 3, *p* < 0.001).

## 4. Discussion

Our study confirms that eggs laid by the same barn swallow females are more similar to each other than to eggs laid by different females. This is consistent with previous findings across various species [11,15,16]. In theory, such individual egg signatures could help swallows distinguish foreign eggs from their own, which would be particularly advantageous in defending against brood parasites or in recognizing their own clutch in dense colonies. The former idea was, however, not supported experimentally because barn swallows have been shown to fail in recognizing and rejecting conspecific or mimetic model eggs placed among their own [60,61]. A similar lack of rejection has been observed in other conspecific brood parasites, such as spotless and common starlings (*Sturnus unicolor* and *Sturnus vulgaris*; [62,63]). In contrast, some hosts of conspecific brood parasites, such as great-tailed grackles (*Quiscalus mexicanus*) and house sparrows (*Passer domesticus*), exhibit relatively high rejection rates of conspecific eggs [64–66]. These contrasting results suggest that egg recognition abilities vary among species, likely owing to differences in how variable eggs are and/or the intensity of selective pressures associated with CBP.

Whether variation in egg appearance helps swallows recognize their own clutches in dense breeding colonies remains an open question. However, we find this also unlikely, as swallows typically engage in nest guarding behaviour and actively chase away intruders [39], including foreign females that could inadvertently attempt to lay eggs in, or incubate, a foreign clutch. Therefore, we believe that the greater variation observed among clutches compared with variation within clutches might be a general phenomenon across bird species, not necessarily driven by selection for egg or clutch recognition. Instead, this pattern likely reflects genetic, developmental, physiological and environmental differences among individual females, which affect the reproductive system, such as shell gland morphology and function. However, the extent to which selective pressures, such as brood parasitism or colonial nesting, contribute to these patterns remains an interesting question for future research.

Interestingly, the identity signal (Hs) was two times lower for swallow eggs compared with that previously observed in common cuckoo eggs [16]. This difference was primarily due to between-clutch variance in cuckoos being more than double that of swallows, suggesting that the cuckoo’s strategy of mimicking the egg phenotypes of various host species likely drives this increased between-clutch variation [67]. This comparison supports the idea that identity signals of bird eggs are strongly influenced by species-specific breeding strategies [21].

Our results show little support for using Eadie’s MED method [23,30] to identify parasitized clutches. Although clutches with MED values exceeding 350 had a 96% likelihood of being parasitized, this approach would fail to detect the majority of parasitized clutches (78%) because their MED values fell below this threshold (figure 3B). We suggest that this may be due to the first- or last-laid egg, which often differs noticeably from other eggs in the clutch, as documented in multiple species [68–70], including barn swallows [40]. Our additional analysis of clutches with known laying sequence of all eggs (*n* = 32) supports this, showing that both the first- and the last-laid eggs differed the most from the others in the clutch (electronic supplementary material). Therefore, we concur with previous studies [41,42], including the research on closely related American cliff swallows (*Petrochelidon pyrrhonota*) [9], and advise against relying solely on egg phenotype to distinguish parasitized from non-parasitized clutches. We suggest including additional data, such as observing two eggs laid on the same day, for more accurate identification.

On the other hand, when parasitism is confirmed in a swallow clutch (e.g. by the appearance of two eggs during the same day), the combination of lower within- and higher between-clutch variation enables relatively accurate identification of parasitic eggs, consistently above the chance. Although human participants performed relatively well in both games, supervised models always achieved higher accuracy (table 2). In the first game, where the task was to identify one parasitic egg among six eggs, error rates remained high (28% for humans and 24% for the model). Therefore, we find assigning the parasitic egg in these situations too unreliable in barn swallows. For other species with lower within-clutch and higher between-clutch variation, it may be possible that the model may yield more reliable results, though this remains to be tested.

**Table 2.** Summary of average identification accuracies in games 1 and 2, achieved by human participants, the unsupervised method and two supervised models.

	game 1 (%)	game 2 (%)
human assessment	71.9	86.8
unsupervised method	62.2	86.5
supervised model: ‘leave-one-clutch-out’	75.7	91.6
supervised model: ‘leave-one-egg-out’	n.a.	97.4

In contrast, in the other scenario, where the choice was between the two eggs laid on the same day (game 2), accuracy was much higher, particularly for supervised models, which made errors only in 8 and 3% of cases (table 2). Hence, we recommend using supervised models, especially the ‘leave-one-egg-out’ approach, for identifying parasitic eggs, but only in the specific case of distinguishing between two eggs laid on the same day. Human assessment, while the easiest method for field researchers, varied across individuals, even among highly experienced researchers. We therefore suggest first testing the performance of individual researchers before relying on their judgement. Consequently, we urge caution when interpreting early studies on CBP in barn swallows that relied solely on human assessment to identify parasitic eggs and laying females [7,39]. This is particularly important in the light of recent findings demonstrating that CBP in this species may be less common than previously believed (a recent study showed only 0.3% parasitism rate [14]). Overall, our results show that once parasitism is detected within a clutch (e.g. through daily nest monitoring of the laying order), supervised machine learning models are currently the most effective approach for identifying parasitic eggs.

We believe the supervised automated method outperformed human participants in identifying parasitic eggs because it analysed a broader range of visual information from the photographs. In contrast, human perception likely prioritizes certain traits over others, limiting the simultaneous processing of multiple traits to the same extent [71,72]. Additionally, the data used in the automated analyses included highly detailed measurements, such as UV reflectance, which is beyond human perception, which may have significantly improved identification accuracy. Indeed, UV spot and background coloration contributed significantly to the second most important variable for egg classification. However, colour data are often strongly positively intercorrelated (e.g. the reflectance of the UV and blue channel [73]), a pattern that also applies to our dataset. Therefore, we expect the supervised model to achieve similar accuracy even when relying only on visible-light photographs. Moreover, the UV coloration of swallow eggs is unlikely to play a role in egg or clutch recognition by swallows themselves, as previous research has shown that UV light barely reaches nests located in buildings [61].

The most informative egg traits for the models were related to egg dimensions—length, width, volume and surface area. These traits were also prioritized by human participants during screen games (personal communication with participants, see also [16]). This is encouraging as it suggests that similar analyses could be effective for species with immaculate eggs, where differences in dimensions and background coloration can still be used. Such an approach could improve parasitic egg detection in waterfowl species where previous studies have failed to achieve sufficient accuracy [25–29]. Interestingly, the random forest model indicated that pattern characteristics were less informative, likely because last-laid swallow eggs tend to have lower maculation [40], reducing the reliability of this trait for egg identification.

In conclusion, the high within-clutch variation in barn swallow eggs prevents reliable identification of parasitized clutches based on egg phenotype alone. However, once parasitism is confirmed (e.g. with unambiguous irregular egg-laying patterns or genetic analyses), supervised automated classification has proven highly effective for identifying the parasitic egg. Since barn swallows have often been cited as a textbook example of conspecific brood parasitism among songbirds, but recent findings suggest they have an extremely low parasitism rate [14], our pipeline may provide a useful approach for a more thorough re-examination of their reproductive behaviour across populations. Beyond barn swallows, we believe that our method can be used to aid in identifying parasitic eggs in other conspecific brood parasites, though its accuracy and the size of the training dataset needed will strongly depend on the egg characteristics of the species.

Identifying the parasitic egg is a crucial first step to enable further studies on host cognition and response toward parasitic eggs, survival of parasitic eggs and chicks (when it is possible to trace which chick hatched from which egg), and, perhaps most importantly, the identification of specific parasitic females by comparing the phenotypes of parasitic eggs with other eggs within a locality [16]. The ability to identify parasitic females directly in the field can open new opportunities to study their behavioural and physiological adaptations, as well as the fitness consequences of conspecific parasitism for both hosts and parasites. To facilitate these broader applications, we have provided a step-by-step markdown guide for our R code, detailing phenotypic traits preparation, model construction and final egg assignment.

Another major strength of automated models resides in their analytical power with larger datasets, while providing more objective assessments compared with human participants, owing to the high level of subjectivity involved within and among individual observers. The main limitation of supervised machine learning models is that they require a labelled dataset with eggs of known identities for training [38], which in our case was achieved through microsatellite genotyping. Nonetheless, supervised models hold great potential for advancing research on avian egg coloration and its links to interesting behaviours such as brood parasitism and colonial nesting.

## Data Availability

All data for these analyses can be found in the supplementary information. Supplementary material is available online [74].
